# Resistance-oriented immunometabolic circuits in laryngeal squamous cell carcinoma: glycolysis–lactate signaling, mitochondrial stress, and tumor–myeloid crosstalk

**DOI:** 10.3389/fimmu.2026.1907469

**Published:** 2026-08-19

**Authors:** Jingjing Sun, Yongxiang Zou, Huimin Yang, Hui Li

**Affiliations:** Department of Otorhinolaryngology Head and Neck Surgery, 960 Hospital of PLA, Ji’nan, China

**Keywords:** glycolysis–lactate axis, laryngeal squamous cell carcinoma, mitochondrial stress, therapeutic resistance, tumor-associated macrophages

## Abstract

Laryngeal squamous cell carcinoma (LSCC) remains clinically challenging because of immune escape and resistance to chemotherapy, radiotherapy, and immune checkpoint blockade. Although immunometabolism encompasses diverse nutrient, redox, and stromal pathways, LSCC-specific evidence is currently strongest for glycolysis/lactate metabolism, mitochondrial remodeling, oxidative stress adaptation, ferroptosis-related regulation, extracellular-vesicle-mediated macrophage remodeling, and checkpoint-associated T-cell dysfunction. This focused review therefore examines resistance-oriented immunometabolic circuits rather than providing an exhaustive catalogue of all metabolic pathways. We discuss how glycolytic activation and lactate accumulation may generate nutrient-competitive and acidic niches; how mitochondrial stress, ROS adaptation, and ferroptosis-related processes influence tumor survival; and how tumor-derived vesicles, TAMs, TILs, Tregs, pDCs, and emerging neutrophil/CAF-related signals shape immune escape. Underexplored axes, including lipid and amino-acid metabolism, glutamine dependence, arginine metabolism, tryptophan–IDO signaling, adenosine metabolism, hypoxia/HIF signaling, NK cells, MDSCs, endothelial cells, and broader stromal–immune interactions, are highlighted as evidence gaps requiring LSCC-specific validation. This review proposes a focused framework for biomarker development and rational combination therapy.

## Highlights

- LSCC immunometabolism is currently best supported by glycolysis–lactate and mitochondrial stress evidence.
- Tumor-derived vesicles, TAM remodeling, and T-cell dysfunction form key suppressive circuits.
- Lipid, amino-acid, IDO, adenosine, MDSC, NK, CAF, and endothelial axes remain evidence gaps.

## 1 Introduction

Laryngeal cancer is an uncommon but clinically important head and neck malignancy with substantial global variation in incidence and mortality (1). According to GLOBOCAN 2022 estimates, approximately 189,200 new cases and 103,400 deaths from laryngeal cancer occurred worldwide, corresponding to an age-standardized incidence rate of 1.9 per 100,000 and an age-standardized mortality rate of 1.0 per 100,000 (2, 3). The burden is markedly sex- and region-dependent: men have substantially higher incidence and mortality than women, and South-Central Asia, Eastern Asia, and Eastern Europe contribute large numbers of cases and deaths, whereas the Caribbean shows the highest age-standardized incidence and mortality rates (4, 5). At the country level, India and China account for the highest numbers of cases and deaths, while Cuba has the highest reported age-standardized incidence and mortality rates (6, 7). Histologically, squamous cell carcinoma represents the dominant subtype of laryngeal cancer, with non-squamous carcinomas accounting for only about 5% of laryngeal malignancies. Therefore, LSCC constitutes the principal pathological entity underlying the global laryngeal cancer burden.

Recent evidence indicates that tumor progression and immune evasion in LSCC are closely linked to metabolic reprogramming within both tumor cells and the tumor immune microenvironment (TIME) (8–11). Metabolic pathways such as glycolysis, oxidative phosphorylation, and lipid metabolism are not merely passive energy sources but actively shape tumor cell behavior and modulate the function of infiltrating immune cells (12, 13). Single-cell transcriptomic analyses have revealed extensive heterogeneity in the LSCC TIME, highlighting distinct immune cell populations and their metabolic states that correlate with tumor progression and patient prognosis (14). Moreover, integrative multi-omics studies have identified immune infiltration phenotypes that vary significantly among LSCC tumors, suggesting that the interplay between metabolic programs and immune cell composition may determine both intrinsic tumor aggressiveness and responsiveness to therapy (15).

Beyond immune heterogeneity, metabolic reprogramming itself contributes to the classification of molecular subtypes of LSCC with divergent prognoses and therapeutic vulnerabilities. Recent analyses have stratified LSCC tumors based on distinct metabolic profiles, revealing subtype-specific enrichment of glycolytic, mitochondrial, or lipid metabolic pathways that are associated with differences in immune infiltration and survival outcomes (16–18). These findings underscore the critical role of immunometabolic crosstalk in shaping tumor behavior and highlight the potential of targeting metabolic vulnerabilities to improve therapeutic efficacy.

Several recent reviews have summarized metabolic reprogramming and immune microenvironment characteristics in laryngeal carcinoma (19, 20). In particular, Ma et al. reviewed glycolysis, lipid metabolism, amino-acid biosynthesis, immunosuppressive tumor microenvironment features, tumor-associated macrophages, T-cell dysfunction, immune checkpoint regulation, and immunotherapeutic strategies in laryngeal carcinoma (10). While this review provides an important overview of metabolic and immune alterations relevant to immunotherapy, substantial conceptual overlap would arise if the present manuscript were framed merely as another general review of LSCC metabolism and immunity. Therefore, the present review is positioned differently. Rather than broadly cataloguing metabolic pathways or immune-cell populations, we focus on resistance-oriented immunometabolic circuits that connect tumor-cell metabolic adaptation with immune-cell dysfunction, exosome-mediated myeloid remodeling, mitochondrial and ferroptosis-related stress, and resistance to chemotherapy, radiotherapy, and immune checkpoint blockade. By distinguishing LSCC-specific evidence from broader head-and-neck cancer concepts, we aim to provide a mechanistic and translational framework for biomarker development, patient stratification, and rational combination therapy.

### 1.1 Evidence classification and critical appraisal strategy

To avoid assigning equal weight to heterogeneous types of evidence, this review classifies the cited studies according to the nature of their supporting data. We distinguish among: 1) transcriptomic, single-cell, multi-omics, or bioinformatic associations; 2) *in-vitro* functional perturbation evidence; 3) *in-vivo* validation; 4) retrospective clinical or tissue-cohort associations; 5) prospective treatment-response evidence; and 6) mechanistic hypotheses proposed by the authors of this review. Bioinformatic signatures and immune-infiltration estimates are interpreted as hypothesis-generating unless supported by functional or clinical validation. *In-vitro* studies are considered mechanistic but model-dependent, whereas *in-vivo* and patient-level treatment-response studies are regarded as stronger translational evidence. When direct LSCC-specific evidence is lacking, we explicitly describe the statement as indirect evidence or as a mechanistic hypothesis requiring further validation.

## 2 LSCC-specific etiological, anatomical, and treatment-related framework

### 2.1 Etiological exposures: tobacco, alcohol, and HPV context

LSCC has distinct etiological features that may influence immunometabolic remodeling. Tobacco and alcohol exposure can induce chronic epithelial injury, oxidative stress, inflammatory signaling, DNA damage, and repeated tissue repair, thereby creating a microenvironment in which metabolic adaptation and immune suppression may co-evolve (21, 22). These exposures may also affect redox metabolism, mitochondrial stress responses, stromal remodeling, and myeloid-cell recruitment. In contrast to oropharyngeal squamous cell carcinoma, where HPV status defines a clinically and immunologically distinct disease subset, the role of HPV in LSCC is less clearly established (23). Therefore, HPV-related immune biology should not be directly extrapolated from oropharyngeal cancer to LSCC without careful stratification.

### 2.2 Anatomical subsite: glottic, supraglottic, and subglottic LSCC

Anatomical subsite is another LSCC-specific determinant that should be considered when interpreting immune and metabolic data. Glottic, supraglottic, and subglottic tumors differ in local tissue architecture, exposure to lymphatic drainage, patterns of spread, and clinical presentation. These differences may influence immune-cell access, spatial immune organization, hypoxia, stromal composition, and the likelihood that sampled tissue captures invasive, immune-excluded, or inflamed tumor regions (24). Supraglottic tumors, for example, are generally more closely linked to lymphatic dissemination than early glottic tumors, which may affect the relationship among macrophage infiltration, lymphangiogenesis, and metastatic risk. Therefore, future LSCC immunometabolic studies should report and analyze tumor subsite whenever possible.

### 2.3 Local hypoxia, cartilage invasion, and metabolic stress

Local hypoxia and cartilage invasion represent clinically relevant features of advanced LSCC that may shape metabolism and immunity. Hypoxic niches can favor glycolytic adaptation, lactate accumulation, acidification, redox stress, and immune exclusion. Cartilage invasion may further reflect a locally aggressive tumor state associated with tissue remodeling, impaired perfusion, stromal activation, and heterogeneous immune-cell access. These features provide a disease-specific context for interpreting glycolysis, mitochondrial stress, macrophage polarization, and treatment resistance in LSCC (25, 26). However, direct studies linking cartilage invasion to spatial immunometabolic gradients remain limited and should be prioritized in future work.

### 2.4 Treatment-related constraints: surgery, radiotherapy, chemoradiotherapy, and organ preservation

Treatment modality also shapes LSCC biology and complicates interpretation of immunometabolic findings. Surgical specimens provide spatially informative tissue but often represent untreated or resectable disease, whereas biopsy samples from patients receiving radiotherapy, chemoradiotherapy, or organ-preservation treatment may capture only limited regions of a heterogeneous tumor (23, 27, 28). Radiotherapy and chemoradiotherapy can alter tumor metabolism, induce oxidative stress, remodel vasculature, modify antigen release, and change immune-cell infiltration. Organ-preservation strategies may also select for resistant tumor-cell states and immune-suppressive niches. Therefore, LSCC immunometabolic biomarkers should be interpreted in relation to sampling time, prior treatment exposure, tumor subsite, and treatment intent.

### 2.5 Working model

Based on these considerations, we propose a disease-specific working model in which LSCC etiology, anatomical subsite, lymphatic drainage, local hypoxia, cartilage invasion, and treatment exposure act as upstream constraints that shape metabolic stress and immune accessibility. These factors may determine whether a tumor develops a glycolytic, lactate-rich, macrophage-enriched, immune-excluded, or checkpoint-high microenvironment. This framework helps distinguish LSCC-specific biology from broader HNSCC immunometabolism and provides a clinical context for interpreting mechanistic and biomarker studies.

## 3 Tumor immune microenvironment in LSCC

### 3.1 Composition and heterogeneity of the TIME

The LSCC TIME should be interpreted in relation to tumor subsite, lymphatic drainage, local invasion pattern, and treatment history. These clinical variables may influence whether immune cells can access tumor nests, remain excluded in stromal regions, or accumulate in lymphangiogenic and hypoxic niches. The tumor immune microenvironment (TIME) in LSCC is highly complex and heterogeneous, comprising multiple immune cell populations that interact with tumor and stromal cells to influence disease progression, therapy response, and patient prognosis (14, 29). Key immune components include tumor-infiltrating lymphocytes (TILs), tumor-associated macrophages (TAMs), regulatory T cells (Tregs), plasmacytoid dendritic cells (pDCs), and other myeloid subsets. Additionally, immune checkpoint molecules, such as PD-1 and PD-L1, as well as various inflammatory cytokines, orchestrate immune evasion and tumor-promoting inflammation (30).

Recent single-cell and multi-omics analyses have provided unprecedented insights into the heterogeneity of LSCC TIME. Qian et al. (2022) demonstrated that LSCC tumors exhibit significant variability in immune cell composition, revealing distinct subpopulations with differing functional states that correlate with patient outcomes (31). Tevetoğlu et al. (2024) further characterized TILs, highlighting the roles of CD3+, CD8+, and FoxP3+ subsets in mediating antitumor immunity and immunosuppressive networks (32). Similarly, Tudor et al. (2024) reported the prognostic significance of PD-L1 expression along with macrophage markers CD68 and CD163, suggesting that specific immune infiltration patterns are tightly linked to tumor aggressiveness. Beyond traditional immune cell types, emerging evidence emphasizes the contribution of pDCs and their ligand-receptor interactions. Ji et al. (2024) revealed that tumor-infiltrating pDCs expressing high levels of PD-L2 modulate CD8+ T-cell activity, potentially promoting immune escape in LSCC (33). TAMs, particularly the CD206+ M2-like subtype, have also been implicated in suppressing antitumor immunity through interactions with CD4+ T cells, reinforcing the complexity of the TIME landscape (34). Low TIL density has been consistently associated with poorer prognosis in early-stage LSCC, underscoring the clinical relevance of immune heterogeneity (35).

Collectively, these studies highlight that LSCC is characterized by a highly heterogeneous TIME, where the relative abundance, spatial distribution, and functional polarization of immune cells significantly influence disease trajectory and patient outcomes. Understanding these patterns is essential for identifying prognostic biomarkers and designing personalized immunotherapeutic strategies.

### 3.2 Immunosuppressive mechanisms within the TIME

The immunosuppressive landscape of LSCC TIME is shaped by multiple mechanisms that impede effective antitumor immunity. Among these, M2-polarized TAMs play a central role by secreting immunosuppressive cytokines, remodeling the extracellular matrix, and interacting with T cells to inhibit their cytotoxic function. Tumor-derived exosomes also contribute to immune escape by delivering molecular signals that reprogram immune cells toward tolerogenic or suppressive states. For example, Chen et al. (2025) showed that STC1-containing small extracellular vesicles derived from LSCC cells reprogram tumor-associated macrophages toward an M2-like phenotype, which subsequently impairs CD8⁺ T-cell function (36). This finding supports a tumor-to-myeloid-to-T-cell signaling axis rather than a pathway initiated by M2-TAM-derived vesicles. Wang et al. (2022) further reported that LSCC-derived exosomal HOTAIR induces macrophage M2 polarization through the PI3K/AKT pathway and promotes EMT and metastasis (37), indicating that tumor-derived extracellular vesicles can remodel macrophage states and facilitate malignant progression. In addition, Su et al. (2022) demonstrated that tumor-derived HMGB1 activates RAGE signaling in M2 macrophages and promotes lymphangiogenesis (38). This study should therefore be interpreted as evidence for HMGB1–RAGE-mediated lymphangiogenic crosstalk involving existing M2 macrophages, rather than direct evidence that HMGB1 induces M2 polarization or CD8⁺ T-cell dysfunction. Xu et al. (2022) linked ferroptosis-related processes to M2 macrophage-derived exosomes in LSCC (39). Wang P et al. (2022) reported that exosomes from M2 macrophages promoted glycolysis by inhibiting PDLIM2 and stabilizing PFKL in FaDu cells (40); because FaDu is a hypopharyngeal carcinoma-derived cell model, this study should be considered indirect model evidence rather than definitive LSCC-specific evidence.

**Figure 1:**
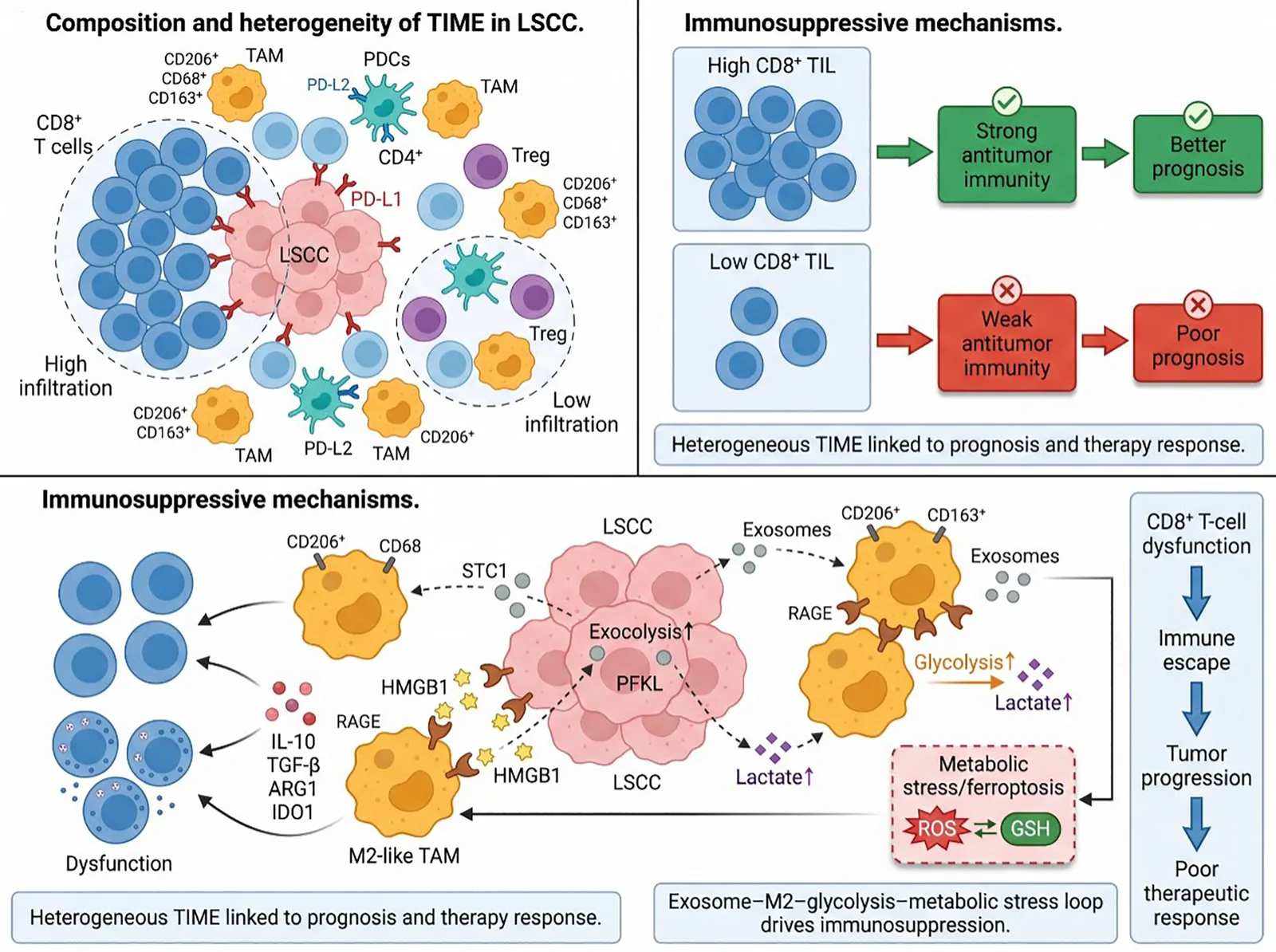
Immunosuppressive tumor immune microenvironment in LSCC. The figure illustrates heterogeneous immune infiltration around PD-L1–expressing LSCC cells, including CD8+ TILs, CD4+ T cells, FoxP3+ Tregs, pDCs, and M2-like TAMs. LSCC-derived exosomes, HMGB1–RAGE signaling, M2-TAM polarization, glycolysis, lactate accumulation, and ferroptosis-related metabolic stress collectively suppress CD8+ T-cell cytotoxicity, promoting immune escape, tumor progression, and poor therapeutic response.

Together, these findings illustrate that LSCC TIME is not only heterogeneous but also dynamically regulated by metabolic and exosome-mediated mechanisms that collectively suppress T-cell cytotoxicity and promote tumor immune escape. Deciphering these pathways provides a foundation for designing interventions that simultaneously target metabolic vulnerabilities and immune checkpoints, thereby enhancing antitumor immunity. **Table 1** summarizes the major immune cell populations and checkpoint molecules present in the LSCC TIME, highlighting their heterogeneity and functional relevance. These immune features are closely linked to prognosis, therapeutic response, and immunosuppressive mechanisms. **Figure 1** summarizes the cellular heterogeneity and immunosuppressive organization of the LSCC TIME. It highlights how M2-TAM polarization, exosome-mediated signaling, and metabolic reprogramming cooperate to impair CD8+ T-cell function and support therapeutic resistance.

**Table 1:** Immune landscape and evidence hierarchy in LSCC TIME.

Immune component	Main LSCC evidence	Evidence stage	What the evidence supports	What the evidence does not support	Interpretation	Reference/PMID
Tumor-infiltrating lymphocytes, TILs (CD3⁺, CD8⁺, FoxP3⁺)	CD3⁺, CD8⁺, and FoxP3⁺ TIL abundance and composition vary among LSCC tumors and are associated with prognosis	Retrospective tissue-cohort/immune-marker association	Supports TIME heterogeneity and candidate prognostic relevance of TIL composition	Does not prove causal immune control of tumor progression or validated prediction of treatment response	TILs should be interpreted as clinically relevant immune-context markers, not definitive predictive biomarkers	(32, 35)
M2-like tumor-associated macrophages, M2-like TAMs (CD206⁺, CD68⁺, CD163⁺)	M2-like macrophage markers are associated with suppressive TIME features and tumor progression-related phenotypes	Tissue-based association/selected mechanistic immune-context evidence	Supports a role for macrophage-enriched immune suppression and tumor–myeloid crosstalk	Does not directly establish clinical immunotherapy resistance unless treatment-response cohorts are analyzed	M2-like TAMs are candidate mediators of immune suppression; therapeutic relevance requires further validation	(34, 41)
Regulatory T cells (Tregs)	Tregs are enriched in suppressive immune contexts and may correlate with reduced effector immune activity	Retrospective immune-marker association	Supports association between Treg infiltration and immune-suppressive TIME	Does not prove direct causality for immune escape or treatment resistance in LSCC	Tregs represent a suppressive immune component requiring spatial and functional validation	(14)
Plasmacytoid dendritic cells, pDCs (PD-L2⁺ pDCs)	PD-L2⁺ pDCs are linked to altered CD8⁺ T-cell infiltration and unfavorable immune context	Tissue-based/immune-context association	Supports a potential pDC-related checkpoint-associated immune-suppressive axis	Does not establish that pDCs independently drive immune escape or predict response to immune checkpoint blockade	pDCs may contribute to checkpoint-associated immune suppression; mechanistic validation is needed	(33)
PD-1/PD-L1 expression	PD-L1 expression is observed on tumor and/or immune cells and is associated with immune-checkpoint activity in LSCC	Tissue-based immune-marker association	Supports checkpoint relevance and possible immune-suppressive signaling in LSCC TIME	Does not by itself prove validated prediction of PD-1/PD-L1 blockade response without treatment-response data	PD-1/PD-L1 should be described as an immune-checkpoint feature and candidate biomarker, not a fully validated predictive marker	
LSCC cell-derived STC1-containing sEVs	STC1-containing sEVs released by LSCC cells reprogram TAMs toward an M2-like phenotype and impair CD8⁺ T-cell function	Mechanistic *in-vitro*/immune functional evidence	Supports a direct LSCC tumor-cell–macrophage–T-cell suppressive axis	Does not establish a clinically tested EV-targeted therapy or validated treatment-response biomarker	One of the stronger direct LSCC examples linking tumor-derived vesicles to immune dysfunction	(36)
LSCC-derived exosomal HOTAIR	LSCC-derived exosomal HOTAIR induces macrophage M2 polarization through PI3K/AKT signaling and promotes EMT/metastasis	Mechanistic *in-vitro*/tumor–macrophage crosstalk evidence	Supports tumor-derived exosomal lncRNA-mediated macrophage remodeling	Does not directly prove CD8⁺ T-cell dysfunction or immunotherapy resistance	Provides direct tumor–macrophage evidence, but downstream T-cell and therapy-response effects require validation	(37)
HMGB1–RAGE signaling in M2 macrophages	Tumor-derived HMGB1 activates RAGE signaling in M2 macrophages and promotes lymphangiogenesis	Mechanistic/tissue-associated evidence	Supports macrophage-associated lymphangiogenic crosstalk	Does not prove HMGB1-induced M2 polarization, CD8⁺ T-cell dysfunction, or immune-checkpoint resistance	Should be interpreted as a lymphangiogenesis-related tumor–macrophage axis, not a broad immune-evasion mechanism	(38)

### 3.3 Underexplored stromal and innate immune compartments

Although TAMs, TILs, Tregs, and pDCs are currently the best-characterized immune compartments in LSCC, other stromal and innate immune populations should be incorporated into future immunometabolic frameworks. Neutrophils are emerging as clinically relevant suppressive cells in LSCC (10, 42, 43). For example, tumor-derived G-CSF/GM-CSF has been reported to prolong neutrophil survival and promote immunosuppression and progression in LSCC, while PD-L1⁺ neutrophils induced by GM-CSF can suppress T-cell function and predict unfavorable prognosis (44). CAF-related stromal remodeling may also influence immune resistance, as a POSTN⁺ CAF/APOE⁺ TAM axis has been associated with tumor progression and potential immunotherapy resistance. However, direct LSCC-specific evidence linking MDSCs, NK-cell metabolic dysfunction, endothelial metabolism, or CAF-derived metabolites to therapeutic resistance remains limited. These compartments should therefore be described as emerging or indirect components rather than established LSCC immunometabolic mechanisms.

## 4 Immunometabolic reprogramming in LSCC

### 4.1 Glycolysis and lactate metabolism

Because glycolysis and lactate metabolism have been extensively summarized in previous LSCC reviews, this section does not aim to repeat a pathway-by-pathway description (45, 46). Instead, we emphasize how glycolytic activation functions as an immunometabolic driver of T-cell dysfunction, TAM polarization, EMT, metastasis, and therapy resistance. Metabolic reprogramming is a defining biological feature of LSCC, with enhanced glycolysis representing one of the most frequently observed metabolic alterations (47–51). Similar to other solid tumors, LSCC cells can preferentially rely on aerobic glycolysis even under oxygen-sufficient conditions, a phenomenon known as the Warburg effect. This metabolic state enables cancer cells to rapidly generate ATP, maintain biosynthetic precursor supply, and adapt to hypoxic or nutrient-limited tumor microenvironments. Importantly, glycolysis is not only a tumor-intrinsic survival mechanism but also a key regulator of the tumor immune microenvironment (18, 52–54). In general cancer immunometabolism, high tumor glucose consumption and lactate accumulation can create nutrient-competitive and acidic niches that impair effector lymphocytes and favor immunosuppressive myeloid phenotypes. However, in LSCC, these immune consequences have not been directly demonstrated for most glycolysis-regulating molecules and should therefore be interpreted as plausible immunometabolic hypotheses rather than established LSCC-specific mechanisms.

Several recent studies have directly linked glycolytic reprogramming to LSCC progression. Zhou et al. reported that ribophorin II (RPN2) promotes LSCC proliferation, migration, epithelial–mesenchymal transition (EMT), and glycolysis, partly through ROS-mediated activation of the PI3K/Akt pathway. This study supports a close coupling between metabolic activation and invasive phenotypes in LSCC (54). Similarly, Li et al. showed that circMYOF facilitates LSCC cell growth, metastasis, and glycolysis through the miR-145-5p/OTX1 regulatory axis, indicating that non-coding RNA networks can remodel tumor metabolism while simultaneously enhancing malignant behavior. Glycolytic reprogramming also appears to contribute to therapeutic resistance (55). Zhang et al. demonstrated that CAPRIN1 enhances both glycolysis and chemoresistance in LSCC via regulation of ZIC5, suggesting that metabolic adaptation may provide energetic and redox advantages that allow tumor cells to survive chemotherapy-induced stress (56). In parallel, Chen et al. found that elevated miR-125b-5p is associated with improved prognosis and suppresses LSCC malignancy and glycometabolic disorder by targeting MAP3K9 (57). Together, these findings indicate that LSCC glycolysis is controlled by diverse molecular regulators, including membrane-associated proteins, circular RNAs, transcriptional regulators, and microRNAs.

The evidence linking glycolytic regulators to LSCC progression is currently strongest at the tumor-cell level. RPN2, circMYOF/miR-145-5p/OTX1, CAPRIN1/ZIC5, and miR-125b-5p/MAP3K9 have been associated with glycolysis, proliferation, EMT, metastasis-related phenotypes, or chemoresistance in LSCC models. However, most of these studies did not directly measure lactate-dependent macrophage polarization, antigen presentation, CD8⁺ T-cell dysfunction, NK-cell activity, or immunotherapy response. Therefore, these molecules should be presented as tumor-intrinsic metabolic regulators with potential immunometabolic relevance, rather than as direct evidence for immune suppression. The proposed glycolysis/lactate–TAM–T-cell axis remains a biologically plausible but incompletely validated mechanism in LSCC.

Accordingly, this review separates three levels of evidence for glycolysis-related immune regulation in LSCC: first, direct LSCC evidence showing tumor-derived extracellular-vesicle-mediated macrophage remodeling and subsequent T-cell dysfunction; second, tumor-cell functional studies showing glycolysis-associated proliferation, EMT, metastasis, or chemoresistance without direct immune assays; and third, broader cancer immunometabolism concepts suggesting that lactate and nutrient competition may impair antitumor immunity. Only the first category should be considered direct LSCC metabolic–immune evidence, whereas the latter two categories are interpreted as indirect evidence or author-proposed hypotheses.

### 4.2 Mitochondrial metabolism and metabolic subtyping

In LSCC, mitochondrial metabolism has been studied primarily in relation to tumor-cell survival, oxidative stress, mitochondrial apoptosis, mitophagy, and treatment adaptation. These studies support a tumor-intrinsic role for mitochondrial remodeling in LSCC progression and therapy response (18, 46). By contrast, direct LSCC-specific evidence showing how mitochondrial metabolism regulates T-cell activation, memory formation, macrophage polarization, or dendritic-cell function remains limited. These immune-cell mitochondrial processes are well established in broader cancer immunometabolism, but their relevance to LSCC should be treated as extrapolated mechanistic context rather than direct LSCC evidence. Therefore, this section distinguishes tumor-intrinsic mitochondrial findings from inferred immune-metabolic consequences.

Recent bioinformatic and multi-omics studies have begun to define the clinical relevance of mitochondrial and broader metabolic programs in LSCC (58). Hu et al. constructed a prognostic model based on mitochondrial metabolism-related genes and reported that this model showed promising performance for predicting LSCC prognosis. This work highlights mitochondrial metabolism-related genes as potential biomarkers for risk stratification and therapeutic decision-making (59). Shen et al. developed a two-gene metabolism-related prognostic risk score based on GPT and SMS. A higher GPT/SMS risk score was associated with poorer overall survival and disease-free survival, supporting its potential value as a candidate prognostic signature (60). However, this study did not define glycolytic versus oxidative metabolic subtypes and did not directly evaluate immune infiltration, immunotherapy response, or drug sensitivity. Therefore, the GPT/SMS model should not be interpreted as evidence for metabolic subtype-specific immune states or therapeutic vulnerabilities. Zheng et al. used transcriptomic, mutational, methylation, and single-cell RNA-sequencing data to define two metabolic subtypes, LCA1 and LCA2, through an independent multigene unsupervised-clustering framework (16). LCA1 was associated with better prognosis, greater metabolic enrichment, and lower immune infiltration, whereas LCA2 was associated with poorer prognosis, higher immune infiltration, and greater T-cell/APC co-inhibition and inhibitory-checkpoint expression. These findings support computational associations between metabolic subtypes and immune-context differences, but they do not establish a binary glycolytic-versus-oxidative classification based on direct metabolic measurements. Prospective validation and functional metabolic assays are still required before these subtypes can be used to guide treatment selection. Importantly, metabolic signatures and computational subtypes can identify candidate prognostic groups and immune-context associations, but they do not by themselves establish causal mitochondrial control of immune-cell function or validated treatment sensitivity. Functional perturbation, spatial metabolomics, and prospective treatment-response studies are required to convert these associations into mechanistic or clinical evidence.

**Figure 2:**
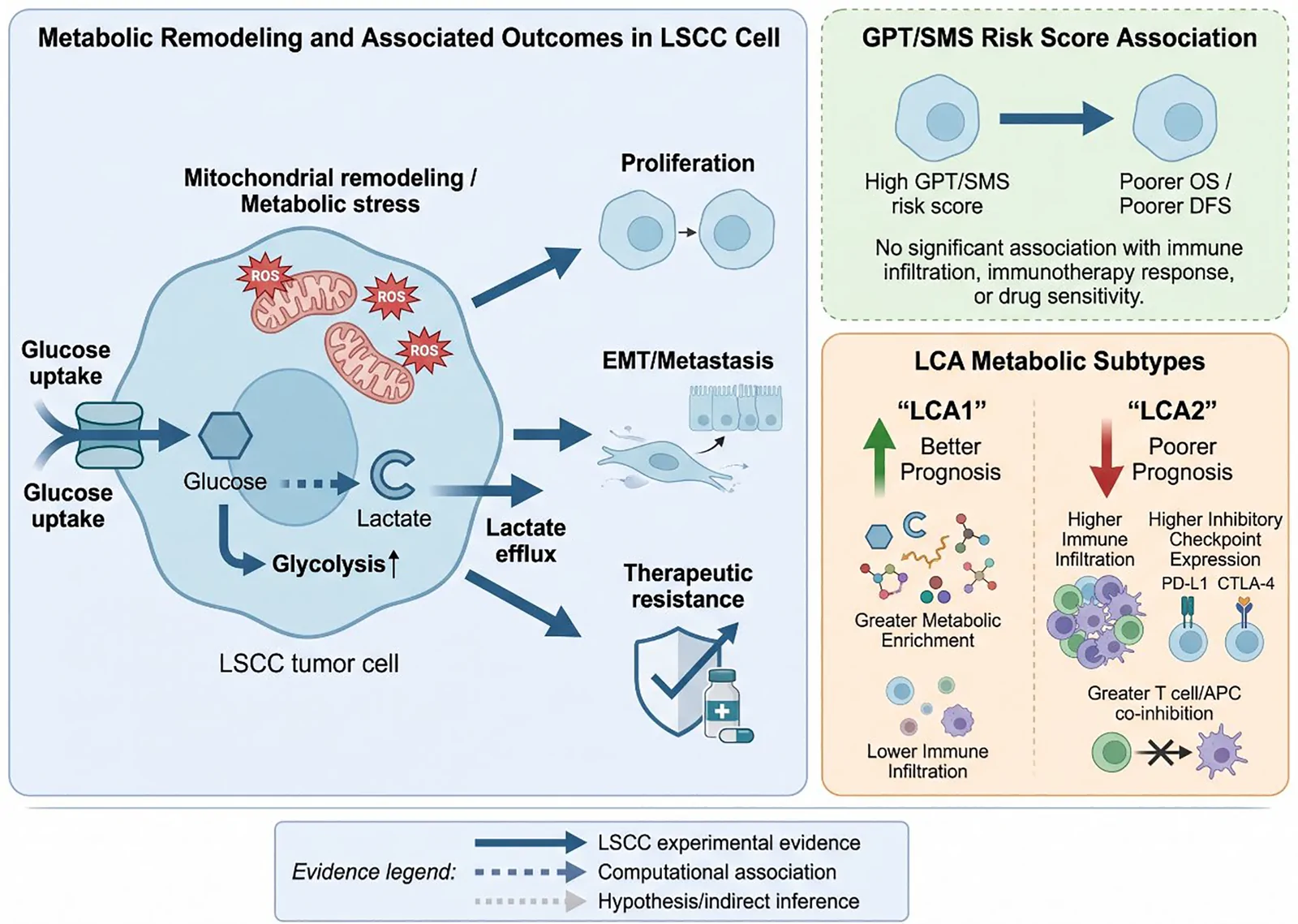
Evidence-stratified overview of metabolic remodeling and metabolic classification in LSCC. Tumor-cell glycolytic and mitochondrial remodeling is supported by LSCC experimental studies involving RPN2, circMYOF/miR-145-5p/OTX1, CAPRIN1/ZIC5, and miR-125b-5p/MAP3K9. The GPT/SMS model should be interpreted only as a two-gene candidate prognostic risk score associated with survival outcomes, not as evidence for immune infiltration, immunotherapy response, or drug sensitivity. LCA1 and LCA2 represent computational metabolic subtypes derived from an independent unsupervised-clustering framework: LCA1 is associated with better prognosis, greater metabolic enrichment, and lower immune infiltration, whereas LCA2 is associated with poorer prognosis, higher immune infiltration, and stronger T-cell/APC co-inhibition and inhibitory-checkpoint expression. Solid arrows indicate LSCC-specific experimental evidence, dashed arrows indicate computational associations, and dotted arrows indicate hypotheses requiring validation.

Overall, mitochondrial metabolism and metabolic subtyping provide a broader framework for understanding LSCC heterogeneity beyond single-gene alterations (53). Future studies should integrate mitochondrial metabolic signatures with single-cell immune profiling, spatial transcriptomics, metabolomics, and clinical treatment-response data. Such integrated approaches may help identify patients with glycolytic, oxidative, immune-inflamed, or immune-suppressed metabolic phenotypes and guide rational combination strategies, such as pairing immune checkpoint blockade with inhibitors of glycolysis, lactate transport, mitochondrial metabolism, or macrophage-mediated immune suppression. **Table 2** provides a concise overview of key metabolic pathways altered in LSCC, including glycolysis, lactate metabolism, and mitochondrial function. It highlights the molecular regulators and their impact on tumor progression, immune modulation, and potential prognostic stratification. **Figure 2** separates experimentally supported tumor-cell metabolic remodeling from computational prognostic signatures and metabolic-subtype associations. This distinction is important because GPT/SMS currently supports only candidate prognostic stratification, whereas LCA1/LCA2 represent computationally defined metabolic subtypes with immune-context associations but without direct metabolic measurement or prospective treatment-response validation.

**Table 2:** Metabolic reprogramming in LSCC: evidence type, immune validation, and interpretation.

Metabolic axis/category	Key regulators or models	Actual evidence in LSCC or related model	Evidence stage	Immune outcome directly measured?	Interpretation	Reference/PMID
Glycolysis/lactate metabolism	RPN2; circMYOF/miR-145-5p/OTX1; CAPRIN1/ZIC5; miR-125b-5p/MAP3K9	These regulators are associated with glycolysis, proliferation, EMT, metastasis-related phenotypes, or chemoresistance in LSCC models	*In-vitro* functional perturbation/tumor-cell phenotyping	No direct measurement of lactate-dependent TAM polarization, antigen presentation, CD8⁺ T-cell cytotoxicity, NK-cell function, or immunotherapy response	Supports tumor-intrinsic glycolytic remodeling; immune consequences should be described as inferred or hypothetical	(54–57)
Mitochondrial and redox metabolism	Mitochondrial metabolism-related genes; oxidative-stress and apoptosis-related programs	Mitochondrial metabolism-related models suggest associations with prognosis, redox homeostasis, apoptosis, and tumor-cell stress adaptation	Computational prognostic association/selected tumor-cell evidence	No direct LSCC-specific evidence showing regulation of T-cell activation, memory formation, macrophage polarization, or dendritic-cell function	Candidate tumor-cell metabolic and prognostic axis; immune-cell effects remain inferential	(59)
Macrophage-to-tumor glycolytic regulation	M2 macrophage-derived exosomes; PDLIM2/PFKL axis	M2 macrophage-derived exosomes promote glycolysis through PDLIM2/PFKL regulation in FaDu cells	Indirect functional model evidence	Partial; macrophage-derived exosomes were studied, but FaDu is not an LSCC-specific model and full immune outcomes were not validated	Suggests possible macrophage-to-tumor metabolic crosstalk; requires LSCC-specific validation	(40)
GPT/SMS prognostic risk score	GPT/SMS two-gene signature	A GPT/SMS-based risk score is associated with overall survival and disease-free survival	Retrospective computational prognostic association	No direct immune functional assay; no validated immunotherapy-response or drug-sensitivity prediction	Candidate prognostic model only; should not be described as a tool for personalized treatment selection	(60)
LCA1/LCA2 computational metabolic subtypes	Multigene metabolic-subtype classification	LCA1 and LCA2 show differences in prognosis, metabolic enrichment, immune infiltration, and inhibitory-checkpoint features	Computational multi-omics association	Immune context inferred computationally; no direct metabolic assay or prospective treatment-response validation	Candidate subtype framework; should not be simplified as glycolytic versus oxidative phenotypes	(16)

### 4.3 Underexplored metabolic axes in LSCC immunometabolism

Beyond glycolysis/lactate metabolism and mitochondrial remodeling, several metabolic programs are central to cancer immunometabolism but remain incompletely characterized in LSCC. Lipid metabolism and ferroptosis-related pathways may influence membrane remodeling, oxidative stress, immune-cell function, and therapy sensitivity, but direct mechanistic links to LSCC immune escape are still limited (61). Amino-acid metabolism is also clinically relevant, as metabolism-related signatures involving GPT/SMS and amino acid-related genes such as SHMT1 have been associated with LSCC prognosis (45). Nevertheless, glutamine dependence, arginine metabolism, tryptophan–IDO signaling, and adenosine metabolism have not yet been systematically validated as LSCC-specific immunometabolic mechanisms. Similarly, hypoxia/HIF signaling, redox metabolism, and nutrient competition are plausible drivers of immune suppression but require spatial, single-cell, metabolomic, and functional validation in LSCC tissues and treatment-response models. These pathways should therefore be considered important research gaps rather than established mechanisms in the current LSCC literature.

## 5 Metabolic reprogramming and therapeutic resistance

A key distinction of the present review is its resistance-oriented interpretation of LSCC immunometabolism. Rather than treating metabolic reprogramming and immune suppression as parallel phenomena, we discuss how glycolysis, lactate accumulation, mitochondrial adaptation, exosome-mediated TAM remodeling, and checkpoint activation converge to generate resistance to chemotherapy, radiotherapy, and immune checkpoint blockade (62–65). Alterations in glycolysis, mitochondrial function, and lipid metabolism can generate adaptive advantages for tumor cells, allowing them to survive treatment-induced stress and evade immune-mediated cytotoxicity. Importantly, these metabolic changes often intersect with immunosuppressive mechanisms, creating a synergistic network that reinforces resistance.

One key mechanism involves the interplay between tumor metabolism and tumor-associated macrophages (TAMs) (44, 66–69). Chen et al. (2025) demonstrated that LSCC cell-derived STC1-containing small extracellular vesicles reprogram TAMs into an M2-like phenotype, thereby impairing CD8⁺ T-cell function (36). This finding indicates that tumor-derived vesicles can initiate a suppressive macrophage–T-cell axis in LSCC. Wang et al. (2022) showed that LSCC-derived exosomal HOTAIR induces macrophage M2 polarization through the PI3K/AKT pathway and promotes EMT and metastasis (37). Separately, Wang P et al. (2022) reported that M2 macrophage-derived exosomes enhanced glycolysis in FaDu cells by inhibiting PDLIM2 and stabilizing PFKL (40). Because FaDu is not an LSCC-specific model, this observation should be used cautiously as indirect evidence for macrophage-to-tumor metabolic crosstalk. Together, these studies support the existence of extracellular-vesicle-mediated crosstalk among LSCC cells, macrophages, and T cells. However, the direct contribution of these pathways to clinical resistance to chemotherapy, radiotherapy, or immune checkpoint blockade remains insufficiently validated and should be investigated in treatment-response cohorts and functional models.

Circular RNAs also participate in this immunometabolic network. Li et al. (2022) showed that circMYOF drives LSCC cell proliferation, metastasis, and glycolysis through the miR-145-5p/OTX1 axis, linking metabolic reprogramming to enhanced tumor aggressiveness and potential chemoresistance (55). By promoting glycolytic flux and lactate production, circMYOF not only fuels tumor growth but also modulates the immune microenvironment to favor immune suppression, highlighting a dual role in both metabolic adaptation and immune evasion. Moreover, intrinsic tumor metabolic regulators can directly influence therapy sensitivity. Zhang et al. (2022) reported that CAPRIN1 enhances both glycolysis and chemoresistance via regulation of ZIC5, suggesting that metabolic activation can provide energetic and redox advantages to withstand cytotoxic stress (56). These findings collectively underscore that metabolic reprogramming in LSCC is tightly linked with therapeutic resistance, forming a feedback loop in which glycolysis, lactate accumulation, and immunosuppressive signals reinforce one another.

**Figure 3:**
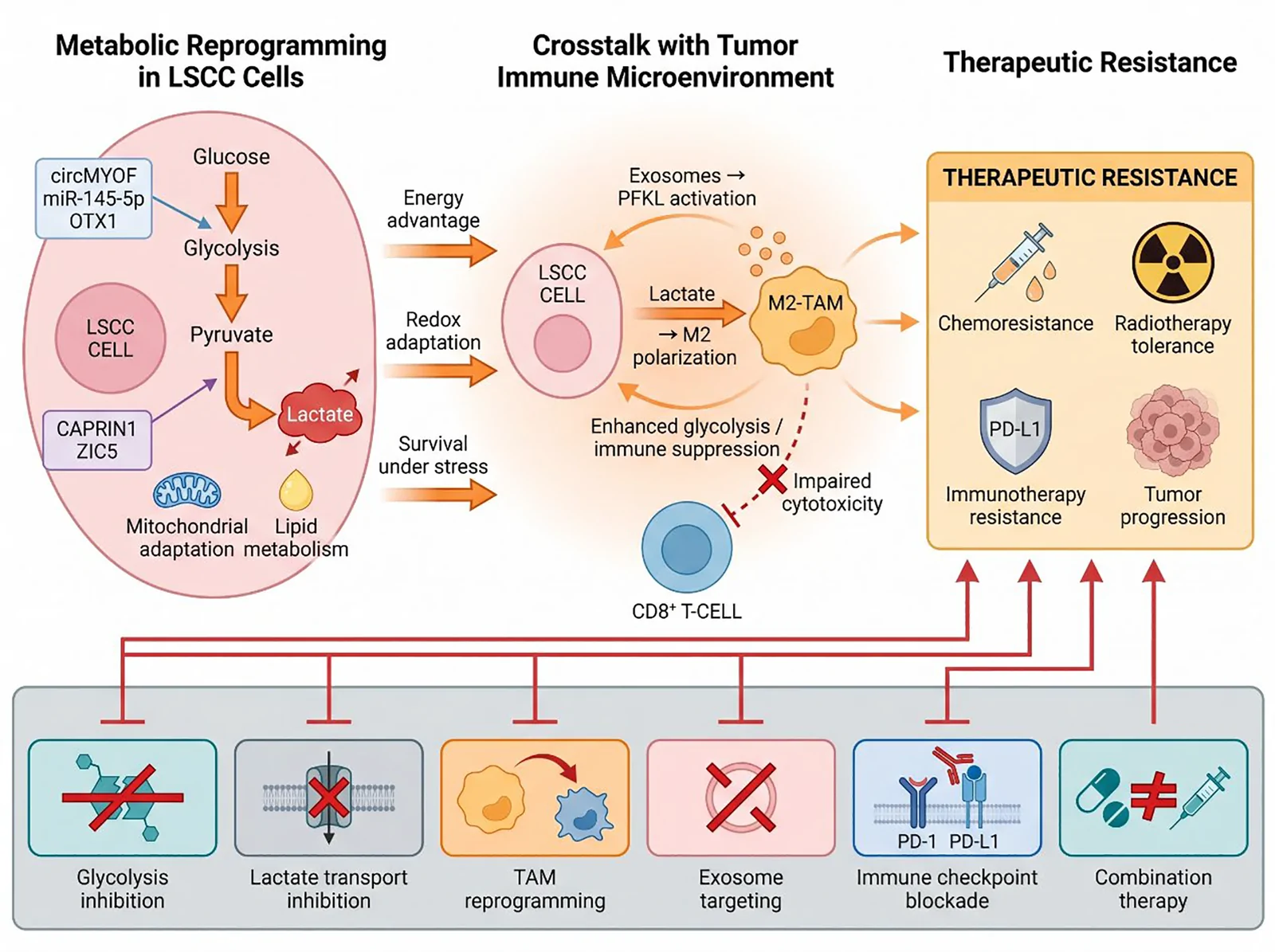
Metabolic reprogramming and therapeutic resistance in LSCC. Enhanced glycolysis, lactate accumulation, and metabolic stress adaptation in LSCC cells cooperate with M2-like tumor-associated macrophages and exosome-mediated signaling to suppress CD8+ T-cell function and reinforce immune escape. This immunometabolic feedback loop drives chemoresistance, radiotherapy tolerance, immunotherapy resistance, and tumor progression, while highlighting potential intervention points such as glycolysis inhibition, TAM reprogramming, exosome targeting, and PD-1/PD-L1 blockade.

In summary, the convergence of metabolic adaptation and immune modulation in LSCC creates a formidable barrier to effective therapy. Exosome-mediated M2-TAM reprogramming, circRNA-driven glycolysis, and intrinsic metabolic regulators collectively promote chemoresistance, radiotherapy tolerance, and immune evasion (70–73). Targeting these immunometabolic circuits—such as combining glycolysis or lactate transport inhibitors with immune checkpoint blockade—represents a promising strategy to overcome therapy resistance and improve clinical outcomes in LSCC. **Table 3** summarizes proposed therapeutic implications of LSCC immunometabolism according to their actual evidence stage. To avoid overstating translational readiness, the table distinguishes *in-vitro* perturbation evidence, indirect model evidence, retrospective computational associations, and author-proposed combination strategies from interventions that have been directly tested in LSCC. **Figure 3** illustrates how tumor-intrinsic metabolic adaptation and macrophage-mediated immune suppression cooperatively drive therapeutic resistance in LSCC. It also highlights the immunometabolic feedback network as a potential target for combination treatment strategies.

**Table 3:** Proposed therapeutic implications of LSCC immunometabolism: actual evidence stage and translational limitations.

Proposed strategy or biomarker	Actual evidence in LSCC or related model	Evidence stage	What the evidence supports	What the evidence does not support	Translational interpretation	PMID
Targeting glycolysis-related tumor-cell regulators	RPN2, circMYOF/miR-145-5p/OTX1, CAPRIN1/ZIC5, and miR-125b-5p/MAP3K9 regulate glycolysis, proliferation, EMT, metastasis-related phenotypes, or chemoresistance in LSCC models	*In-vitro* functional perturbation/tumor-cell phenotyping	Glycolysis-related regulators contribute to LSCC malignant behavior; CAPRIN1/ZIC5 supports a link with chemoresistance	Does not demonstrate that glycolysis inhibition restores T-cell function, reverses immune suppression, or improves immunotherapy response	Candidate tumor-cell metabolic targets; immune-restorative effects remain hypothetical	(54–57)
Targeting mitochondrial or redox metabolism	Mitochondrial metabolism-related models and selected mitochondrial apoptosis/redox studies suggest links with prognosis, oxidative stress, apoptosis, or treatment adaptation	Computational prognostic model/selected tumor-cell functional evidence	Mitochondrial and redox programs may contribute to LSCC prognosis, tumor-cell survival, apoptosis, and therapy-related stress responses	Does not identify a validated mitochondrial-targeted agent, LSCC immune model, or therapeutic experiment showing improved clinical response	Candidate research direction requiring defined agents, LSCC-specific models, immune assays, and treatment-response validation	(59)
Blocking tumor-derived extracellular-vesicle signaling	LSCC cell-derived STC1-containing small extracellular vesicles reprogram TAMs toward an M2-like phenotype and impair CD8⁺ T-cell function; LSCC-derived exosomal HOTAIR induces M2 polarization and promotes EMT/metastasis	Mechanistic *in-vitro*/tissue-associated evidence	Tumor-derived vesicles can remodel macrophage states and may contribute to CD8⁺ T-cell dysfunction or malignant progression	Does not establish a clinically tested anti-exosome therapy in LSCC	Proposed strategy to disrupt tumor–macrophage–T-cell crosstalk; currently preclinical	(36, 37)
Targeting macrophage-to-tumor metabolic crosstalk	M2 macrophage-derived exosomes promote glycolysis through the PDLIM2/PFKL axis in FaDu cells	Indirect functional model evidence	Supports a possible macrophage-to-tumor glycolytic regulation mechanism	FaDu is a hypopharyngeal carcinoma-derived model; this does not prove LSCC-specific therapeutic benefit	Indirect evidence; requires validation in LSCC-specific cell lines, organoids, animal models, and patient tissues	(40)
Metabolic intervention plus PD-1/PD-L1 blockade	Current LSCC studies support the relevance of PD-1/PD-L1, PD-L2, TAMs, TILs, and suppressive immune cells, but do not directly test metabolic inhibition plus checkpoint blockade	Author-proposed translational hypothesis/indirect rationale	Provides biological rationale for future combination studies	Does not demonstrate synergy between metabolic inhibitors and PD-1/PD-L1 blockade in LSCC	Testable hypothesis requiring animal validation and prospective treatment-response studies	(33, 36)
GPT/SMS prognostic risk score	A two-gene GPT/SMS risk score is associated with poorer overall survival and disease-free survival	Retrospective computational prognostic association	Candidate prognostic stratification	Does not define glycolytic versus oxidative subtypes; does not predict immune infiltration, immunotherapy response, drug sensitivity, or personalized treatment selection	Candidate prognostic biomarker only; not a validated predictive tool	(60)
LCA1/LCA2 computational metabolic subtypes	Multigene unsupervised clustering identified LCA1 and LCA2 with different prognosis, metabolic enrichment, immune infiltration, and inhibitory-checkpoint features	Computational multi-omics association	Candidate metabolic subtype classification and immune-context association	Does not establish direct metabolic measurements, binary glycolytic-versus-oxidative classes, or validated treatment-response prediction	Useful for hypothesis generation; requires functional and prospective validation	(16)

## 6 Translational opportunities

### 6.1 Biomarkers and prognostic signatures

The immunometabolic landscape of LSCC offers multiple avenues for the identification of clinically relevant biomarkers and prognostic signatures (45, 74, 75). Integrating immune infiltration patterns with metabolic gene expression allows for a more precise stratification of patients based on risk and potential therapeutic response. Metabolic gene signatures reflecting glycolysis, mitochondrial function, or lactate metabolism should currently be regarded as candidate prognostic biomarkers rather than validated predictive markers of treatment response.

Shen et al. (2022) developed a metabolism-related gene signature based on GPT and SMS genes, demonstrating that this signature can robustly predict LSCC prognosis by capturing tumor metabolic heterogeneity (60). Similarly, Hu et al. (2025) constructed a prognostic model centered on mitochondrial metabolism-related genes, which not only stratified patient survival outcomes but also highlighted the functional relevance of mitochondrial oxidative phosphorylation and energy homeostasis in tumor progression (59). Zheng et al. (2025) further characterized LSCC metabolic subtypes using multi-omics approaches, revealing that distinct metabolic profiles are associated with differential immune infiltration and clinical outcomes (16). Collectively, these studies suggest that metabolic biomarkers may support future patient stratification. However, their use for guiding personalized therapy remains investigational because prospective validation in treatment-response cohorts is still lacking.

Beyond prognostication, combining immune and metabolic metrics provides opportunities for dynamic monitoring of treatment response. For instance, patients with high glycolytic activity and immunosuppressive TIME may benefit from therapies targeting both metabolic pathways and immune checkpoints, emphasizing the translational relevance of these integrated signatures.

### 6.2 Potential therapeutic strategies

Translationally, the insights gained from LSCC immunometabolic research provide a rationale for targeting metabolic vulnerabilities to enhance therapy efficacy. One approach is to directly modulate tumor metabolism. Glycolysis inhibitors, lactate transport blockers, or mitochondrial-targeted agents can potentially disrupt the energetic and biosynthetic support required for tumor growth and immune evasion (76–79). By reducing lactate accumulation or restoring nutrient availability in the TIME, these strategies may enhance effector T-cell activity and reverse immunosuppression (80–84). At present, however, most of these therapeutic concepts remain preclinical or hypothetical in LSCC. Direct prospective evidence showing that metabolic inhibitors improve immune checkpoint blockade, chemotherapy, or radiotherapy responses in LSCC patients is not yet available. Targeting the immunometabolic crosstalk between tumor cells and the microenvironment represents another promising avenue. Chen et al. (2025) demonstrated that STC1-containing small extracellular vesicles released by LSCC cells reprogram TAMs toward an M2-like state and consequently impair CD8⁺ T-cell function (36). This suggests that targeting tumor-derived vesicle signaling may help attenuate macrophage-mediated T-cell suppression. Wang et al. (2022) reported that LSCC-derived exosomal HOTAIR induces M2 polarization through the PI3K/AKT pathway and promotes EMT and metastasis (37), supporting exosomal lncRNA signaling as a potential target for disrupting tumor–macrophage communication. By contrast, evidence that M2 macrophage-derived exosomes directly enhance glycolysis comes from a FaDu cell model through the PDLIM2/PFKL axis (40), and should be described as indirect rather than LSCC-specific evidence. Su et al. (2022) showed that tumor-derived HMGB1 activates RAGE signaling in M2 macrophages and promotes lymphangiogenesis (38), suggesting a potential lymphangiogenic axis but not directly proving HMGB1-induced M2 polarization, CD8⁺ T-cell dysfunction, or immune escape.

Combining metabolic intervention with PD-1/PD-L1 blockade is a biologically plausible strategy, but direct LSCC-specific evidence remains lacking. Current LSCC studies support the relevance of immune checkpoints, TAM remodeling, T-cell dysfunction, and selected metabolic regulators; however, they do not yet demonstrate that glycolysis inhibition, lactate transport blockade, mitochondrial targeting, or exosome inhibition synergizes with checkpoint blockade (85–87). Therefore, such combinations should be presented as testable translational hypotheses rather than established therapeutic strategies. Moreover, strategies that reprogram TAMs or modulate exosome secretion may further enhance the efficacy of immunometabolic therapies, creating a multi-pronged approach to target the complex crosstalk within the LSCC microenvironment. Despite their mechanistic appeal, EV- or exosome-blocking strategies remain difficult to translate clinically. Most available evidence is derived from *in-vitro* or mechanistic models, whereas systemic inhibition of EV secretion or uptake *in vivo* may lack tumor specificity. Because EVs participate in normal immune regulation, tissue repair, vascular communication, and intercellular homeostasis, broad blockade may cause unintended biological effects. In addition, EV-targeting agents must overcome pharmacokinetic barriers, including tumor accumulation, tissue penetration, systemic clearance, dosing schedule, and off-target distribution. These challenges are particularly relevant in LSCC, where tumor subsite, vascularity, hypoxia, stromal architecture, and prior radiotherapy or chemoradiotherapy may further affect drug delivery. Therefore, future studies should evaluate EV-targeting approaches in LSCC-specific organoids, animal models, pharmacodynamic assays, and treatment-response cohorts before clinical translation can be considered.

## 7 Critical appraisal of current evidence and evidence gaps

Overall, the current LSCC immunometabolism literature is characterized by an imbalance between hypothesis-generating molecular studies and treatment-response evidence. Transcriptomic, single-cell, and multi-omics studies have been useful for identifying immune phenotypes, metabolic subtypes, and candidate prognostic signatures, but they remain largely correlative (88, 89). *In-vitro* perturbation studies provide stronger mechanistic support for specific regulators of glycolysis, EMT, chemoresistance, ferroptosis, or macrophage remodeling, but they are limited by cell-line dependence and incomplete modeling of the native TIME. *In-vivo* validation remains relatively sparse, and prospective clinical evidence linking immunometabolic biomarkers to response to chemotherapy, radiotherapy, or immune checkpoint blockade is especially limited. Therefore, several connections proposed in this review—such as glycolysis/lactate-driven T-cell dysfunction, mitochondrial stress-mediated immune escape, and metabolic inhibition combined with checkpoint blockade—should be viewed as mechanistic hypotheses or translational opportunities rather than established clinical strategies.

## 8 Future perspectives

Despite significant advances in understanding the immunometabolic landscape of LSCC, many critical questions remain unresolved. Emerging technologies now provide unprecedented opportunities to dissect tumor metabolism and immune interactions at high resolution, offering insights that may guide precision therapy and biomarker development (90–94). One promising direction is the integration of single-cell transcriptomics with multi-omics datasets, including genomics, epigenomics, metabolomics, and proteomics. Such approaches allow the identification of distinct tumor cell subpopulations and immune subsets, revealing how metabolic programs influence immune cell function and contribute to intratumoral heterogeneity. Sun et al. (2024) demonstrated the utility of single-cell transcriptomic analysis in LSCC, uncovering extensive TIME heterogeneity and identifying specific immune populations associated with aggressive phenotypes and therapy resistance (14). By coupling these data with metabolic profiling, researchers can begin to map the precise crosstalk between tumor metabolism and immune regulation, which is essential for predicting patient-specific treatment responses. Future studies should explicitly integrate LSCC-specific clinical metadata into immunometabolic analyses. Single-cell, spatial, and metabolomic studies should stratify samples by glottic, supraglottic, or subglottic subsite; smoking and alcohol exposure; HPV status; cartilage invasion; nodal status; prior treatment exposure; and organ-preservation versus surgical treatment. Spatial approaches are particularly important for mapping hypoxic, lactate-rich, lymphangiogenic, immune-excluded, and cartilage-invasive niches within LSCC tissues.

Spatial transcriptomics and mass spectrometry imaging represent another frontier in LSCC research. These technologies enable high-resolution mapping of metabolic and immune landscapes within the native tumor architecture, providing critical insights into how localized metabolic niches affect immune cell recruitment, activation, and suppression (88, 95–97). Zheng et al. (2025) highlighted that metabolic subtypes in LSCC correlate not only with global gene expression patterns but also with differences in immune cell infiltration and functional states (16). Incorporating spatial context into metabolic-immune analyses can therefore reveal microenvironmental constraints that may underlie therapeutic resistance and identify region-specific targets for intervention. Future spatial studies should move beyond descriptive mapping and test LSCC-specific spatial hypotheses. Hypoxic or necrotic tumor cores may harbor stronger glycolytic activity, lactate accumulation, mitochondrial stress, and myeloid-dominant immune suppression, whereas well-vascularized invasive margins may show greater immune-cell access, antigen-presentation activity, and tumor–immune interactions. Cartilage-invasive or poorly perfused regions may represent metabolically stressed and immune-excluded niches that are underrepresented in superficial biopsies. In treatment-exposed LSCC, radiotherapy or chemoradiotherapy may further generate spatially restricted resistant niches characterized by hypoxia, stromal remodeling, suppressive myeloid accumulation, and exhausted or excluded CD8⁺ T cells. Therefore, future studies should integrate spatial transcriptomics, metabolite imaging, multiplex immunofluorescence, and clinical metadata to map hypoxic cores, invasive margins, lymphovascular regions, cartilage-invasive fronts, and post-treatment resistant areas.

Finally, translating these mechanistic insights into the clinic remains a major challenge. Prospective clinical trials are needed to evaluate the efficacy of interventions targeting immunometabolic circuits, including glycolysis inhibitors, mitochondrial metabolism modulators, and strategies to reprogram suppressive immune populations. Integrating metabolic biomarkers and immune signatures as predictive tools may enable stratification of patients who are most likely to benefit from such combination therapies. Shen et al. (2022) demonstrated that metabolism-related gene signatures, such as GPT/SMS, can effectively predict LSCC prognosis, supporting the feasibility of implementing metabolic markers in clinical decision-making (60).

In summary, future research in LSCC should focus on high-resolution, multi-dimensional characterization of immunometabolic networks, integration of spatial context, and clinical validation of targeted interventions. These efforts will be critical for translating basic discoveries into therapies capable of overcoming treatment resistance and improving outcomes for patients with LSCC.

## 9 Conclusion

Immunometabolic reprogramming has emerged as a central mechanism shaping the biological behavior, immune landscape, and therapeutic response of LSCC. Rather than functioning as separate processes, tumor metabolism and immune regulation are tightly interconnected. Enhanced glycolysis, lactate accumulation, mitochondrial metabolic remodeling, and altered metabolic gene expression can support tumor proliferation, epithelial–mesenchymal transition, metastasis, and resistance to therapy. At the same time, these metabolic changes reshape the TIME by influencing immune-cell infiltration, macrophage polarization, T-cell dysfunction, immune checkpoint expression, and inflammatory signaling. The heterogeneity of the LSCC TIME further complicates disease progression and treatment response. Different immune cell populations, including tumor-infiltrating lymphocytes, M2-like tumor-associated macrophages, regulatory T cells, plasmacytoid dendritic cells, and PD-1/PD-L1–related immune subsets, contribute to distinct immune states with different prognostic implications. Importantly, immunosuppressive mechanisms are often reinforced by metabolic adaptation. Exosome-mediated communication, macrophage reprogramming, glycolytic activation, and ferroptosis-related metabolic stress collectively generate a microenvironment that favors immune escape and therapeutic resistance. From a translational perspective, immunometabolic features provide promising opportunities for biomarker discovery and therapeutic innovation. Metabolism-related gene signatures, mitochondrial metabolism models, and computational immune-metabolic subtypes may help identify candidate prognostic groups and immune-context differences in LSCC. However, they should not yet be considered validated predictors of treatment sensitivity. Therapeutically, targeting glycolysis, lactate transport, mitochondrial metabolism, exosome signaling, or TAM polarization may improve responses to chemotherapy, radiotherapy, and immune checkpoint blockade. Such combination strategies are particularly attractive because they address both tumor-intrinsic metabolic survival programs and microenvironment-mediated immune suppression. Nevertheless, the field remains at an early stage. Most current evidence is based on retrospective cohorts, bioinformatic analyses, limited experimental validation, or small clinical datasets. Future studies should integrate single-cell sequencing, spatial transcriptomics, metabolomics, proteomics, and functional experiments to define causal immunometabolic circuits in LSCC. Prospective clinical studies are also needed to validate immunometabolic biomarkers and test rational combination therapies. A deeper understanding of LSCC immunometabolism may ultimately enable more precise patient stratification and support the development of personalized therapeutic strategies capable of overcoming treatment resistance and improving clinical outcomes. By repositioning LSCC metabolism and immunity within a resistance-oriented circuit framework, this review extends beyond descriptive summaries of metabolic pathways or immune-cell populations and highlights clinically actionable connections among metabolic adaptation, immune escape, and treatment failure.
